# Dynamic Mode Decomposition-Based Clustered Pattern Projection for Reliable Alzheimer’s Disease Detection from EEG

**DOI:** 10.3390/diagnostics16040530

**Published:** 2026-02-10

**Authors:** Jong-Hyeon Seo, Hunseok Kang, Jacob Kang, Aymen I. Zreikat

**Affiliations:** 1 School of Basic Sciences, Hanbat National University, Daejeon 34158, Republic of Korea; hyeonni94@hanbat.ac.kr; 2 College of Engineering and Technology, American University of the Middle East, Egaila 54200, Kuwait; aymen.zreikat@aum.edu.kw; 3 College of Computer, Mathematical, and Natural Sciences, University of Maryland, College Park, MD 20742, USA; jkang115@umd.edu

**Keywords:** electroencephalography (EEG), Alzheimer’s disease (AD), dynamic mode decomposition (DMD), eyes-open (EO), leave-one-subject-out cross-validation (LOSO), clustered pattern projection (CPP), margin-based reliability

## Abstract

**Background/Objectives:** Detecting Alzheimer’s disease (AD) from normal aging using eyes-open (EO) EEG is challenging due to stimulus-driven nonstationarity and fragmented oscillatory responses. This study aims to determine whether prototype-based representations derived from Dynamic Mode Decomposition (DMD) can improve AD detection from EO photostimulation EEG. **Methods:** We propose a DMD-based framework termed DMD-based Clustered Pattern Projection (DMD-CPP). Segment-wise DMD representations were clustered to learn class-specific medoid prototypes, and each EEG epoch was encoded as a vector of cosine-similarity coordinates with respect to these prototypes. A linear SVM classifier was trained on the resulting DMD-CPP features and evaluated under strict leave-one-subject-out validation. **Results:** The DMD-CPP model achieved competitive classification accuracy and, importantly, enhanced margin-based reliability. In EO photostimulation, AD versus healthy control classification showed a pronounced improvement, characterized by wider and more asymmetric decision margins, particularly assigning low confidence to normal epochs misclassified as AD. Tasks involving frontotemporal dementia also showed improvement, although the effect was less pronounced than for AD. **Conclusions:** Clustering-based pattern projection has been shown to stabilize EEG dynamics and provide an interpretable, confidence-aware feature representation. These findings suggest that DMD-CPP offers a promising framework for reliable AD detection from EO EEG, where conventional spectral methods typically struggle.

## 1. Introduction

Alzheimer’s disease (AD) and frontotemporal dementia (FTD) are major neurodegenerative disorders that are associated with heterogeneous and overlapping cognitive and behavioral manifestations, particularly in early disease stages, which complicates reliable clinical assessment [1,2,3,4], while structural and metabolic imaging modalities such as MRI and FDG-PET are well established [5,6], they are costly and limited in temporal sensitivity to functional neural changes. Electroencephalography (EEG), by contrast, provides a low-cost and temporally precise measure of large-scale neural dynamics and has been widely investigated as a complementary biomarker for dementia [7,8,9].

Most prior EEG-based studies on dementia have focused on the eyes-closed (EC) condition, where relatively stable posterior alpha rhythms enable reliable spectral characterization [10,11]. Under EC, AD-related alterations are often characterized by reproducible changes in oscillatory power, coherence, or complexity, implicitly assuming the existence of disease-specific patterns that are sufficiently stable to be captured by averaging or covariance-based representations [12]. In this setting, a wide range of methods—including power spectral density (PSD) features, entropy-based complexity measures, and Riemannian geometry-based covariance representations—have reported promising classification performance for AD and FTD [10,13,14]. More recently, deep learning approaches such as convolutional neural networks (CNNs) and Transformers have further improved accuracy under EC conditions [12,15,16,17].

However, this assumption does not readily extend to eyes-open (EO) conditions. Under EO stimulation, posterior alpha rhythms are suppressed, and stimulus-driven responses become highly nonstationary. In AD, visual entrainment is often fragmented and unstable, reflecting impaired large-scale synchronization and deficient alpha reactivity [18,19]. FTD exhibits different but partially overlapping alterations, further complicating discrimination [20]. As a result, conventional short-window spectral summaries that perform well under EC conditions degrade substantially under EO, and only a small number of studies have attempted EO-based dementia classification [21]. The recently released OpenNeuro dataset ds006036, recorded under EO photic stimulation, highlights both the diagnostic potential and the methodological challenges of this setting.

A further challenge concerns evaluation methodology. It is now well recognized that EEG classifiers evaluated under non-subject-wise splits can severely overestimate performance. When re-evaluated under strict leave-one-subject-out (LOSO) validation, many models—including deep neural networks—exhibit pronounced performance degradation and even near-zero accuracy for certain subjects [22,23]. Recent large-scale reanalyses have shown that even LOSO accuracy can be misleading, as apparent generalization may collapse under repeated or nested subject-wise validation (e.g., N-LOSO), revealing poor robustness across unseen individuals [22]. These findings underscore that accuracy alone is insufficient for assessing clinical reliability in heterogeneous populations such as AD and FTD.

A recent study has demonstrated that, under EC conditions, carefully designed feature representations—particularly those based on the Riemannian geometry of covariance matrices—can achieve reliable subject-wise performance even under strict LOSO-CV validation, despite limited dataset sizes [14]. These findings indicate that, when disease-related EEG patterns are relatively stable, appropriate feature construction can partially compensate for data scarcity and support generalization across subjects. In such EC settings, reliability is largely driven by the degree to which an individual subject’s representation aligns with characteristic low-frequency connectivity patterns.

Under EO conditions, however, reliability assessment faces an additional challenge beyond classification accuracy. Because subject-level representations are less likely to conform to stable, disease-specific templates, misclassifications often arise from qualitatively unstable or fragmented neural responses rather than marginal deviations from a well-defined pattern. As a result, reliability in EO settings becomes inherently asymmetric: incorrect predictions may not correspond to low-confidence samples near a decision boundary but instead reflect subjects whose neural dynamics fundamentally diverge from the learned structure. This property motivates the need for reliability analyses that explicitly characterize miss-class behavior, rather than relying solely on pattern similarity or distance-based confidence measures.

Meanwhile, a complementary line of research has shifted attention away from the exclusive choice of individual features or classifiers toward the organization of the feature space itself. Rather than treating each EEG epoch as an independent sample to be directly classified, these approaches first cluster extracted representations to identify representative patterns and subsequently encode new samples by their similarity or projection onto these learned prototypes. Such prototype-based or dictionary-based representations have been explored in EEG analysis through Bag-of-Words models, codebook learning, and medoid-based clustering, demonstrating improved robustness to inter-subject variability and enhanced interpretability compared with direct feature classification [24,25,26,27].

Building on this perspective, we propose a white-box EEG framework that explicitly targets the challenges of EO dementia analysis under strict LOSO evaluation. Building on Dynamic Mode Decomposition (DMD), which captures spatio-temporal neural dynamics beyond stationary spectral content [28], we introduce a clustering-based pattern projection approach. Rather than discarding unstable or fragmented responses, the proposed method clusters DMD-derived representations from training data to identify recurring dynamical motifs—including characteristic patterns of breakdown—and encodes each epoch by its similarity to medoid-based prototypes. In this way, heterogeneous and nonstationary EO responses are transformed into a structured representation that emphasizes geometric alignment with group-level dynamical patterns rather than raw spectral magnitude.

To address these challenges, we propose a unified framework for EEG-based AD detection that integrates (i) DMD-based long-term summarization of temporally segmented EEG, (ii) a Bag-of-Words-inspired clustering and pattern projection scheme using medoid prototypes to capture recurring disease-relevant dynamics, and (iii) reliability-aware subject-wise evaluation with margin-based analysis to assess generalization under strict LOSO validation. This integrated approach, termed the DMD–CPP (Dynamic Mode Decomposition–Clustered Pattern Projection) framework, is designed to provide an interpretable and generalizable representation of nonstationary EEG dynamics while mitigating the overestimation of performance commonly observed in deep learning-based models.

The main contributions of this work are threefold. First, we address the underexplored problem of EO-based AD discrimination by interpreting the characteristic breakdown of AD responses not as noise but as a recurrent dynamical structure. Using DMD and clustering, these breakdown patterns are consolidated into representative prototype groups and modeled through a projection-based representation. Second, we adopt strict LOSO validation and demonstrate that subject-level reliability cannot be inferred from accuracy alone, motivating the need for confidence-aware analysis. Third, we introduce a margin-based reliability assessment that reveals how the proposed DMD-based clustering framework improves confidence, separability, and controllability at the subject level, particularly for AD-related decisions.

## 2. Materials and Methods

Details of the dataset, feature construction, classification protocol, and experimental setup are provided in this section. The EO photostimulation EEG dataset is introduced, including subject demographics and the inclusion/exclusion criteria, by which ten stimulus-related epochs per subject are obtained. DMD is applied to 2 s slices, and the resulting mode-magnitude representations are temporally summarized within each epoch using simple statistical descriptors to construct fixed-length mode-based feature vectors. To assess reliability beyond nominal accuracy, a margin-based analysis is performed to compare absolute decision margins between correctly classified and misclassified epochs under LOSO validation. For fair comparison, a baseline pipeline based on principal component analysis (PCA) [29] is included. The baseline is evaluated alongside the proposed *DMD-CPP* method, in which mode-based representations are combined with class-specific clustering and projection to enable robust EEG classification.

### 2.1. Dataset

The publicly available EO photostimulation EEG dataset on OpenNeuro (dataset ID: ds006036, v1.0.4; DOI:10.18112/openneuro.ds006036.v1.0.4 (https://openneuro.org/datasets/ds006036/versions/1.0.4, accessed on 5 February 2026)) was analyzed. The dataset includes 88 individuals (36 AD, 23 FTD, 29 Normal Control (CN)), recorded with 19 scalp electrodes (10–20 system; Fp1, Fp2, F3, F4, C3, C4, P3, P4, O1, O2, F7, F8, T3, T4, T5, T6, Fz, Cz, Pz) at 500 Hz (10 µV/mm). During clinical acquisition, intermittent photic stimulation was administered in nominal 5 Hz increments (5, 10, 15, 20, and—where tolerated—up to 30 Hz); however, the ordering, dwell times, and upper limits were allowed to vary across subjects and recording blocks under routine conditions. Detailed event annotations are provided in the dataset’s release files. Cognitive and neuropsychological functioning was assessed using the international Mini-Mental State Examination (MMSE), which yields scores from 0 to 30, with lower values reflecting greater cognitive impairment. The Alzheimer’s disease (AD) cohort comprised 36 participants (11 males, 25 females) with a mean age of 66.2 years (SD = 7.5) and a mean MMSE score of 17.4 (SD = 4.6). The frontotemporal dementia (FTD) group included 23 participants (12 males, 11 females), with an average age of 64.4 years (SD = 7.3) and a mean MMSE score of 22.6 (SD = 2.7). The cognitively normal (CN) group consisted of 29 individuals (17 males, 12 females), with a mean age of 68.3 years (SD = 4.9); all CN participants achieved the maximum MMSE score of 30. All experimental procedures were conducted in accordance with ethical standards and were approved by the Scientific and Ethical Committee of the Aristotle University of Thessaloniki and AHEPA University Hospital (protocol no. 142/12-04-2023).

Although the dataset provides both raw and preprocessed EEG recordings, many prior studies have relied on the preprocessed signals, which already incorporate noise filtering and artifact removal (see [21]). Following this convention, we used the preprocessed EEG data in the present study. Accordingly, no additional preprocessing was applied during feature extraction, as the provided signals were already suitable for analysis. Moreover, because our objective was to examine brain responses elicited by visual stimulation, the analysis was restricted to EEG segments corresponding to visual stimulus events. For each subject, time intervals during which visual stimuli were presented were identified, and only the EEG data within these intervals were extracted for subsequent analysis.

As shown in Table 1, usable durations vary across subjects. Each epoch uses 20 s (10 consecutive 2 s segments). To construct ten epochs per subject, we require at least 19 non-overlapping 2 s segments (i.e., ≥38 s of usable stimulus-related data). Recordings shorter than this threshold were excluded. Note that, unlike EC resting, our EO recordings include visually driven activity (photic entrainment) mixed with spontaneous fluctuations and small ocular/motion events. Segments from different EO stimulation frequencies are therefore analyzed together to assess whether diagnostic patterns generalize beyond any single stimulation condition, rather than reflecting EC-style resting-state dynamics.

Because photic-stimulation frequencies differ across subjects, 20 s epochs may contain mixed frequency segments. A category-based sensitivity analysis reported in [28] found no association between epoch-level stimulus composition and diagnostic group. Accordingly, stimulation was treated as a nuisance factor, justifying a uniform slice-based DMD pipeline (see Appendices 6.1 and 6.2 in [28]).

### 2.2. Feature Extraction

This subsection introduces the construction of fixed-size, mode-based descriptors of stimulus-related EEG that preserve sensor-space structure across time. Each recording was partitioned into non-overlapping 2 s segments; epochs were defined as 20 s windows comprising 10 consecutive segments, with start indices uniformly spaced across subjects, so that each subject contributed 10 uniformly distributed epochs. DMD was applied to every 2 s segment to obtain rectified mode matrices together with their associated eigenfrequencies. Within each epoch, mode-magnitude matrices were aggregated across segments and summarized using temporal statistics (element-wise mean), yielding fixed-length representations suitable for subsequent pattern learning and classification.

The overall pipeline proceeds as follows: Stage 1 constructs mode-based feature vectors from 20 to s epochs by aggregating DMD mode magnitudes across time; Stage 2 learns compact, class-specific pattern dictionaries via hierarchical clustering and medoid selection; finally, each sample is mapped to cosine-similarity features for downstream classification.

#### 2.2.1. Dynamic Mode Decomposition

DMD is a data-driven method that represents multichannel signals as superpositions of coherent spatio-temporal modes with characteristic eigenvalues [30]. In EEG data, where the number of channels is typically much smaller than the number of temporal samples, an extended (stacked) DMD formulation is adopted to enrich the state representation by concatenating consecutive time samples. Under this formulation, the signal admits the modal expansion

(1) $$x\left(t\right)\approx \sum_{j}c_{j}\;\phi_{j}\;e^{\omega_{j}t},$$

where x(t) denotes the multichannel EEG signal at time *t*, $\phi_{j}$ are the DMD modes, $\omega_{j}$ are the corresponding continuous-time eigenvalues, and $c_{j}$ are the modal amplitudes determined by the initial condition. Details of the augmented DMD construction are provided in Appendix A.1.

#### 2.2.2. DMD Configuration and Post–Processing

From the stimulus-related EEG in Table 1, each recording was partitioned into non-overlapping 2 s segments, and one epoch was defined as a sequence of ten consecutive segments (20 s). Let $N_{\mathrm{seg}}$ denote the number of usable 2 s segments for a subject. To ensure uniform coverage of the stimulus interval while avoiding overlap at the segment level, ten epochs were placed at evenly spaced start indices over the range $\left[1,\;N_{\mathrm{seg}}-10+1\right]$:

$$s_{i}=1+\left\lfloor \frac{\left(i-1\right)\;\left(N_{\mathrm{seg}}-10\right)}{9}\right\rfloor ,\;\mathrm{epoch}\;i:\;\left[\;s_{i},\;s_{i}+9\;\right],\;\;i=1,\ldots ,10.$$

This requires $N_{\mathrm{seg}}\geq 19$ (i.e., at least 38 s of usable data); recordings below this threshold were excluded (IDs: 15, 21, 64, 65, and 78; see Table 1). Avoiding overlap is particularly important in stimulus-locked analyses, as overlapping windows can introduce spurious entrainment and related confounds [31,32].

**Figure 1:**
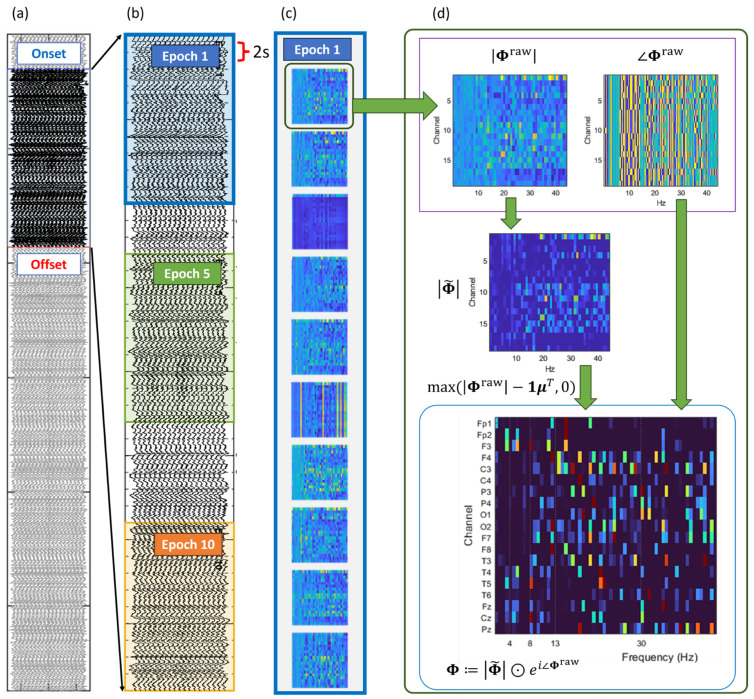
EEG segmentation and DMD-based feature construction pipeline. (**a**) The full EEG recording of subject 10 with the photic-stimulation interval (onset = 15 s, offset = 93 s) highlighted. (**b**) The highlighted interval is divided into non-overlapping 2 s segments and grouped into ten 20 s epochs (only Epochs 1, 5, and 10 are shown). (**c**) DMD is computed once per 2 s segment; epoch panels collect those segment-level results without recomputation. (**d**) For each segment, DMD is applied to the stacked state. From the augmented modes, only the physical *M*-channel portion is extracted to form $\Phi^{\mathrm{raw}}$, and after the volume-conduction correction, the rectified DMD mode matrix Φ is obtained.

As illustrated in Figure 1, DMD was performed once for each non-overlapping 2 s segment within the stimulus onset–offset interval. Epochs served solely as an aggregation and indexing layer: each 20 s epoch collected the ten segment-level DMD feature maps falling within its span. Consequently, although epoch windows may visually overlap, no additional DMD computation was introduced. For each segment, DMD was applied using the stacked formulation with a fixed stacking parameter and truncation size, yielding a finite set of dynamic modes associated with eigenfrequencies $\left\{\omega_{j}\right\}$ as defined in (1). The resulting channel-level DMD modes are collected as

(2) $$\Phi =\left[\;\phi_{1},\;\phi_{2},\;\ldots \;\right],$$

where each $\phi_{j}$ represents the spatial pattern of the *j*-th dynamic mode across the M=19 EEG channels. The truncation size of the reduced-order approximation determines the total number of retained modes. To mitigate sensor-uniform contributions and volume-conduction-related inflation, the DMD modes Φ in (2) were further refined by removing sensor-uniform components within each mode while preserving the inter-sensor phase relationships. This refinement suppresses stimulus-locked harmonic contamination without altering the relative phase structure across EEG channels. Unless stated otherwise, Φ henceforth denotes the rectified DMD mode matrix used in all subsequent analyses. Figure 1d schematically summarizes this step within the overall epoch-level processing pipeline.

The rectification process, which recovers the original *M*-channel representation from the augmented DMD modes, is described in Appendix A.2. For completeness, we note that the formulation admits complex-valued DMD modes with phase information as a general representation to enable future phase-aware extensions. In this study, however, we focus on magnitude-based descriptors to emphasize the spatial participation patterns of the modes, and phase information is therefore not used in the experiments.

#### 2.2.3. Stage 1—Epoch-Level Descriptor Construction

Each 20 s epoch is first partitioned into T=10 non-overlapping segments of length 2 s. For each segment, DMD is applied to the multichannel EEG, yielding a set of rectified DMD modes with associated eigenfrequencies. Only modes with frequencies in the interval [4,40]Hz are retained, thereby excluding very low- and very high-frequency components.

From the retained modes of each segment, a non-negative mode-image is constructed by stacking channel-wise magnitudes and rescaling the mode dimension to a fixed width *P* via interpolation. Here, *P* denotes the number of normalized mode indices used to represent the frequency-ordered DMD spectrum on a common grid. This results in a sequence of rectangular matrices, one per segment, that encode how strongly each EEG channel loads onto the ordered DMD modes. These segment-level representations are then aggregated across the *T* segments within an epoch by computing their elementwise mean, thereby summarizing epoch-level mode activity. Finally, the aggregated representation is linearly rescaled to the unit interval, yielding the epoch-level descriptor

(3) $$\hat{D}\in {\left[0,1\right]}^{M\times P},$$

where *M* denotes the number of EEG channels and *P* denotes the dimensionality of the mode axis after interpolation (set to P=50 in our experiments).

Let ${\hat{D}}_{n}$ denote the epoch-level descriptor of the *n*-th epoch, as defined in (3). Each descriptor is converted into a representation by column-wise stacking,

(4) $$z_{n}=\mathrm{vec}\left({\hat{D}}_{n}\right)\in R^{MP}.$$

Let I denote an index set specifying a subset of epochs; the corresponding *design matrix* is defined as

(5) $$Z_{I}=\left[\;z_{n}:n\in I\;\right],$$

where each column $z_{n}$ in (4) represents the vectorized epoch-level descriptor. This matrix serves as the input to clustering and dictionary (basis) learning. The specific construction of the index set I—for example, according to a training split, a diagnostic group, or a validation protocol—is described separately in the subsequent section.

#### 2.2.4. Stage 2—Basis Learning from the Design Matrix

In Stage 2, basic learning is performed on collections of epoch-level descriptors selected according to a given criterion. Let I denote an index set specifying a subset of epochs, and let $Z_{I}$ be the corresponding design matrix defined in (5).

Hierarchical clustering is a classical approach for identifying structure in high-dimensional data through recursive partitioning based on pairwise dissimilarities [25]. In this work, we employ hierarchical divisive clustering with complete linkage under cosine dissimilarity as a mechanism for selecting representative basis elements from the design matrix $Z_{I}$. Specifically, each column of $Z_{I}$ corresponds to a vectorized epoch-level descriptor, denoted by $z_{i}$. Similarity between two such descriptors, $z_{i}$ and $z_{j}$, is measured using cosine similarity,

$$c_{ij}=\frac{{\left(z_{i}\right)}^{⊤}z_{j}}{∥z_{i}{∥}_{2}\;{∥z_{j}∥}_{2}},\;\delta_{ij}=1-c_{ij},$$

and complete linkage defines the dissimilarity between two subsets as the maximum pairwise dissimilarity between their elements [33]. Based on this criterion, a hierarchical tree is constructed and recursively partitioned from the top level.

Recursive partitioning proceeds by splitting a subset only when it satisfies minimum support and separation conditions, ensuring that the resulting groups correspond to coherent regions of the descriptor space rather than noise-driven artifacts. Specifically, a subset is further divided only if

(a) Its size exceeds a support threshold $\tau_{\sup}$;
(b) The corresponding dendrogram split height exceeds $h_{\mathrm{stop}}$, and;
(c) Both resulting subsets contain at least $n_{\min}$ samples.

The subsets of I that satisfy these criteria are retained as surviving groups,

(6) $${\left\{I_{k}\right\}}_{k=1}^{K},$$

which define candidate regions for representative basis selection.

From each surviving group $I_{k}$ in (6) obtained by hierarchical partitioning, a single representative descriptor is selected as the medoid. Under cosine dissimilarity, the medoid is defined as the sample that minimizes the total dissimilarity to all other members of the group,

$$m_{k}=\arg\min_{i\in I_{k}}\sum_{j\in I_{k}}\delta_{ij},\;b_{k}=z^{\left(m_{k}\right)},$$

where k=1,2,…,K. Collecting the selected medoids yields the dictionary

(7) $$B=\left[\;b_{1},b_{2},\ldots ,b_{K}\;\right]\in R^{MP\times K},$$

where each column of B corresponds to a representative descriptor selected from one surviving group.

The medoid-based selection is adopted instead of a centroid-based alternative for two reasons. First, the medoid corresponds to an actual observed epoch, ensuring that each basis element represents a physically realizable EEG pattern rather than a virtual average. Second, medoids are inherently robust to outliers and skewed cluster geometries, which commonly arise in high-dimensional mode-based descriptors [34,35].

Given a vectorized epoch descriptor z in (4) and the dictionary B defined in (7), the relationship between z and the *k*-th dictionary atom (medoid) is quantified by their cosine similarity,

(8) $$f_{k}\left(z\right)=\frac{z^{⊤}b_{k}}{∥z{∥}_{2}\;{∥b_{k}∥}_{2}},\;k=1,\ldots ,K.$$

The resulting value $f_{k}\left(z\right)$ constitutes the *k*-th component of the dictionary-based feature vector associated with z.

### 2.3. Classification

We employ support vector machines (SVMs) for supervised classification [36]. Because the mode-based representation described in Stages 1–2 is high-dimensional, we adopt the linear SVM as our primary classifier (All classifiers are implemented using MATLAB (R2025b) with the fitcsvm function using a linear kernel.) Linear SVMs provide a robust margin-based baseline, require no kernel-scale tuning, and remain stable in the small-*N*, large-*p* regime—where *N* denotes the number of training samples and *p* the dimensionality of the feature space—typical of subject-level EEG studies.

**Figure 2:**
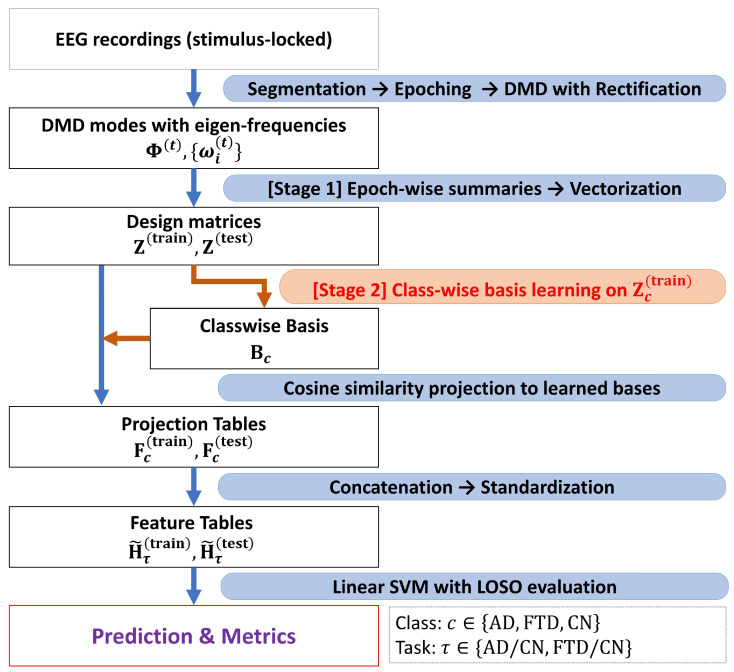
Overall pipeline of the proposed DMD-CPP framework. Rectangular boxes represent intermediate data products or outputs at each stage, whereas rounded boxes indicate processing steps applied between them. Arrows denote the data flow through the pipeline. The orange rounded box and red arrows/text highlight the proposed algorithmic components, namely the class-wise pattern clustering based on the class-wise training design matrices $Z_{c}^{\left(\mathrm{train}\right)}$ as in (10), and the subsequent basis learning that yields the class-specific medoid (basis) dictionaries $B_{c}$ as in (11). Here, $Z^{\left(\mathrm{train}\right)}$ and $Z^{\left(\mathrm{test}\right)}$ denote the design matrices for the training and test sets, respectively, as defined in (9); $F_{c}$ represents the class-specific projection tables as in (12), and ${\tilde{H}}_{\tau }$ denotes the final standardized feature tables for task τ as in (13).

Throughout this work, all learning steps are restricted to training subjects only; no information from the test subjects is used in dictionary learning, normalization, or classifier training. Figure 2 summarizes the complete feature–assembly and classification workflow.

#### 2.3.1. Classification Procedure

Fix a binary classification task τ (AD vs. CN, FTD vs. CN or AD vs. FTD) with the class set

$$Y_{\tau }=\left\{c_{1},c_{2}\right\}.$$

Stage 1 and Stage 2 together provide, for each epoch *n*, a mode-based vectorized descriptor $z_{n}\in R^{MP}$ as in (4), and the split-wise design matrices, defined as instances of the general sub-design matrix in (5),

(9) $$Z^{A}=\left[\;z_{n}:n\in I^{A}\;\right],\;A\in \left\{\mathrm{train},\mathrm{test}\right\},$$

where $I^{A}$ denotes the index set of epochs assigned to split A (with $I^{\mathrm{train}}\cap I^{\mathrm{test}}=⌀$). For a given class $c\in Y_{\tau }$, let $I_{c}^{\left(\mathrm{train}\right)}\subseteq I^{\left(\mathrm{train}\right)}$ denote the index set of training epochs belonging to class *c*. The corresponding class-restricted design matrix is given by

(10) $$Z_{c}^{\left(\mathrm{train}\right)}=\left[\;z_{n}:n\in I_{c}^{\left(\mathrm{train}\right)}\;\right],$$

which serves as the input to the hierarchical divisive clustering procedure. The resulting surviving groups yield medoids and the class-specific dictionary

(11) $$B_{c}\in R^{MP\times K_{c}}$$

as defined in (7). This dictionary learning step is carried out once per class $c_{1}$ and $c_{2}$, using training data only, and is shared by both the training and test evaluations.

Given a dictionary $B_{c}$ and a vectorized descriptor z, its projection onto $B_{c}$ is computed by cosine similarity according to (8), yielding a $K_{c}$-dimensional feature vector for that sample. Collecting these projections over all samples in the split A∈{train,test} produces the feature block

(12) $$F_{c}^{A}\in R^{\left|I_{A}\right|\times K_{c}},\;c\in Y_{\tau }.$$

For task τ, the Stage 2 representation is obtained by concatenating the two class-specific blocks:

$$H_{\tau }^{A}=\left[\;F_{c_{1}}^{A}\;\;F_{c_{2}}^{A}\;\right]\in R^{\left|I_{A}\right|\times \left(K_{c_{1}}+K_{c_{2}}\right)},\;A\in \left\{\mathrm{train},\mathrm{test}\right\}.$$

Thus, $H_{\tau }^{A}$ is the sole input to the downstream classifier for task τ.

To ensure comparability across subjects and prevent scale bias, each feature dimension is standardized using statistics from the training split only. Let μ and σ denote the per-feature mean and standard deviation of $H_{\tau }^{\mathrm{train}}$. We apply the affine normalization

(13) $${\tilde{H}}_{\tau }^{\left(\mathrm{train}\right)}=\frac{H_{\tau }^{\left(\mathrm{train}\right)}-\mu }{\sigma },\;{\tilde{H}}_{\tau }^{\left(\mathrm{test}\right)}=\frac{H_{\tau }^{\left(\mathrm{test}\right)}-\mu }{\sigma }.$$

A linear SVM is trained on ${\tilde{H}}_{\tau }^{\left(\mathrm{train}\right)}$ using the labels in $Y_{\tau }$, and test predictions are obtained by applying the trained classifier to ${\tilde{H}}_{\tau }^{\left(\mathrm{test}\right)}$. All reported metrics (accuracy, precision, recall, F1, and confusion matrices) follow the validation protocol described in the next subsection.

#### 2.3.2. Validation Methodology, Classification Tasks, and Metrics

To rigorously assess generalization while preventing subject-specific leakage, we employ a LOSO validation strategy. In each fold, one subject *s* is held out for testing, and all epochs of *s* are excluded from training. The model is trained on the remaining subjects and evaluated once on the held-out subject. This process is repeated until every subject in the task has served as the test fold.

An important consideration in EEG analysis is that subjects often differ substantially in recording length. As a result, shorter recordings yield a smaller number of epochs with a higher degree of overlap, which undermines the statistical independence of epoch-level samples and introduces asymmetric bias across subjects. For this reason, epoch-level performance metrics may be unreliable and should not be interpreted as reflecting true generalization at the subject level.

Despite this limitation, epoch-level results are reported in parallel for indirect comparison with prior EEG studies that adopt non-subject-wise validation schemes, such as leave-*N*-segments-out or epoch-level cross-validation. In contrast, all primary analyses, statistical evaluations, and interpretations of the proposed method—including the discussion of reliability and generalization—are based exclusively on subject-level summaries, which constitute the clinically and methodologically meaningful unit of analysis.

For each task, predictions from all LOSO folds are pooled by summing the subject-specific confusion matrices. This aggregated epoch-level confusion matrix contains the total ${\mathrm{TP}}_{\mathrm{tot}}$, ${\mathrm{FP}}_{\mathrm{tot}}$, ${\mathrm{TN}}_{\mathrm{tot}}$, and ${\mathrm{FN}}_{\mathrm{tot}}$ counts accumulated over the entire evaluation. Because each subject contributes the same number of test epochs, simple summation ensures a correct and unbiased aggregation.

All reported metrics—accuracy, precision, recall, and F1—are computed exclusively from the aggregated confusion matrix. For example,

$$\mathrm{Precision}=\frac{{\mathrm{TP}}_{\mathrm{tot}}}{{\mathrm{TP}}_{\mathrm{tot}}+{\mathrm{FP}}_{\mathrm{tot}}},\;\mathrm{Recall}=\frac{{\mathrm{TP}}_{\mathrm{tot}}}{{\mathrm{TP}}_{\mathrm{tot}}+{\mathrm{FN}}_{\mathrm{tot}}},$$

with F1 defined analogously.

In addition to epoch-level performance, we evaluated diagnostic performance at the subject level, which represents the clinically relevant unit under LOSO validation. For each test subject, the trained classifier produces multiple epoch-wise predictions. These predictions are aggregated by majority voting to yield a single subject-level decision. A subject is considered correctly classified if more than half of its test epochs are assigned to the true diagnostic class.

Subject-level accuracy is defined as the proportion of subjects whose aggregated predictions match their ground-truth labels. Under LOSO validation, each subject contributes exactly one binary outcome (correct or incorrect), and the resulting accuracy estimate therefore follows a binomial sampling model. To quantify the statistical uncertainty associated with the finite number of subjects, we report 95% confidence intervals for subject-level accuracy using the Wilson score method.

We evaluate three binary discrimination tasks under LOSO: AD vs. CN, FTD vs. CN, and AD vs. FTD. In all tasks, the positive class is defined as AD for AD vs. CN, FTD for FTD vs. CN, and AD for AD vs. FTD.

### 2.4. Decision-Margin Analysis

To assess model reliability beyond accuracy, we performed a decision-margin analysis based on the decision scores produced by the linear SVM. For each test epoch under LOSO evaluation, the classifier outputs a signed decision margin *m*, defined as the distance to the separating hyperplane. The magnitude |m| is commonly interpreted as a measure of prediction confidence [35,37].

#### 2.4.1. Subject-Level Margin Aggregation

Because clinical decisions are made at the subject level, epoch-wise margins were first summarized within each subject. For a given subject *s*, let $m_{s,e}$ denote the signed margin of epoch *e*. Subject-level descriptors were obtained by aggregating these epoch-wise margins within each subject. Specifically, the confidence magnitude of subject *s* was defined as the median of the absolute epoch-wise margins, ${\mathrm{median}}_{e}\left(\right|m_{s,e}\left|\right)$. All subsequent analyses are performed on these subject-level summaries rather than on individual epochs.

#### 2.4.2. Outcome Grouping and Margin Descriptors

Subjects were grouped according to their classification outcome into four categories: true positive (TP), false negative (FN), true negative (TN), and false positive (FP). For each outcome group, subject-level margin behavior was characterized using three complementary descriptors, each computed by first aggregating epoch-wise margins within subjects and then comparing the resulting subject-level summaries across groups.

1. Confidence magnitude (within-subject). For each subject, confidence magnitude was defined as the median of the absolute margins across epochs, ${\mathrm{median}}_{e}\left(\right|m_{s,e}\left|\right)$. This quantity summarizes the typical distance of that subject’s epoch-wise representations from the decision boundary. Group-level comparisons were then performed on these subject-level confidence magnitudes.
2. Within-subject dispersion. For each subject, variability of epoch-wise margins was quantified using the interquartile range ${\mathrm{IQR}}_{e}\left(m_{s,e}\right)$. This descriptor captures how stable or fluctuating the subject’s margins are across epochs. Group-level differences were assessed by comparing these subject-level IQR values across outcome groups.
3. Within-subject sign consistency. For each subject, we computed the proportion of epochs with positive margins, $P_{e}\left(m_{s,e}>0\right)$, and summarized directional stability using the index $|P_{e}\left(m_{s,e}>0\right)-0.5|$. Values close to 0 indicate frequent sign changes across epochs, whereas values approaching 0.5 indicate that the subject’s epoch-wise margins consistently favor one decision direction. These subject-level indices were subsequently compared across outcome groups.

#### 2.4.3. Statistical Analysis

The proposed framework constructs projection features anchored to disease-related break patterns learned during clustering. Under this design, subjects whose data genuinely exhibit such break patterns are expected to show systematically different margin behavior from those that do not. In particular, subjects correctly identified as patients (TP) and those incorrectly projected as patients (FP) are expected to differ in their subject-level margin characteristics, as the latter reflect spurious or unstable matches to the learned disease anchors. Accordingly, our primary interest is to assess whether meaningful differences exist between these outcome groups, with comparisons between TN and FN considered complementary.

To this end, for each subject-level margin descriptor, we tested the null hypothesis that the two outcome groups do not differ in their central tendency. Group differences were summarized using the median difference, Δmed, which captures the separation between the typical subject-level values of the two groups. Statistical significance was assessed using permutation tests, in which subjects were randomly reassigned between the two groups while preserving the original group sizes. This procedure evaluates whether the observed median difference is larger than would be expected by chance alone, under the assumption that group membership carries no systematic information about the descriptor.

All tests were two-sided and conducted at a significance level of α=0.1, reflecting the exploratory nature of subject-level reliability analysis under limited and imbalanced sample sizes. In addition to statistical significance, effect size was quantified using Cliff’s δ, a nonparametric measure of stochastic dominance that estimates the probability that a randomly selected subject from one group has a larger descriptor value than a randomly selected subject from the other group, minus the reverse probability. Cliff’s δ ranges from −1 to 1, with values near zero indicating substantial overlap between groups and larger absolute values indicating stronger separation [38].

### 2.5. Comparing Algorithm: PCA-Based Mode Features

Our main pipeline combines mode-based Stage 1 summaries with class-specific dictionary learning via hierarchical clustering and medoid extraction, followed by classification using a linear SVM. To isolate and assess the contribution of this Stage 2 design, we construct a simpler baseline that employs the same Stage 1 mode-based representation but replaces the clustering-based dictionary learning with PCA. Importantly, both pipelines use the same linear SVM classifier so that any performance differences can be attributed specifically to the choice of feature projection—clustering-based basis learning versus PCA—rather than to differences in the classifier itself.

#### 2.5.1. Shared Mode-Based Stage 1 Representation

The PCA-based baseline uses the same Stage 1 features as the main pipeline. For each 20 s epoch, DMD is applied to the 2 s segments, and the resulting vectorized epoch-level descriptor $z\in R^{MP}$ is constructed as in (3). For a given binary classification task τ with class set $Y_{\tau }=\left\{c_{1},c_{2}\right\}$, we construct task-specific design matrices separately for the training and test splits. Let A∈{train,test} denote the data split, and let $I^{A}$ be the index set of all subjects assigned to split A under LOSO validation. From this set, we select only those subjects whose labels belong to $Y_{\tau }$, yielding the task-restricted index set $I_{\tau }^{A}\subseteq I^{A}$.

The resulting task-specific design matrix is defined as

(14) $$Z_{\tau }^{A}=\left[\;z_{n}:n\in I_{\tau }^{A}\;\right],$$

where each column corresponds to the vectorized descriptor of a subject belonging to one of the two classes in task τ.

#### 2.5.2. PCA-Based Dimensionality Reduction

For each binary task τ, PCA is fitted using only the training design matrix $Z_{\tau }^{\left(\mathrm{train}\right)}$ in (14) to avoid information leakage. After mean-centering, principal components are retained to explain 95% of the total variance, yielding a low-dimensional linear subspace that captures the dominant variation in the mode-based representations. Both training and test samples are then projected onto this task-specific subspace using the same projection learned from the training data.

Following projection, each retained component is standardized using statistics estimated exclusively from the training split, and the same normalization parameters are applied to the test split. This procedure ensures a fair comparison with the proposed clustering-based pipeline by preserving identical data splits, classifiers, and normalization rules while differing only in the choice of feature projection method.

### 2.6. Experimental Setup

All postprocessing, feature extraction, and classification were implemented in MATLAB. This section summarizes the experimental design, including the construction of analysis epochs, the feature-extraction settings, and the evaluation protocol.

#### 2.6.1. Epoch Construction

Each subject’s EEG was segmented into 20 s epochs, each consisting of ten consecutive non-overlapping 2 s segments extracted from stimulus intervals. Under the LOSO scheme, we constructed ten epochs per subject. This epoch length and count reflect a trade-off: longer or more numerous epochs increase discriminability but reduce the number of usable subjects, whereas shorter or fewer epochs yield less reliable representations. We therefore adopted an intermediate setting balancing performance and generalization.

#### 2.6.2. Feature Settings

Feature-extraction hyperparameters were fixed a priori and tuned independently of the downstream classification task. DMD was applied to each segment using a stacked formulation with stack size S=48 and truncation rank R=100, as in (A1). These parameters were selected to balance spectral resolution, numerical stability, and computational tractability; a detailed rationale for these choices is provided in Appendix A.1. The resulting DMD modes were summarized into fixed-size epoch-level descriptors with mode-axis resolution P=50 in (3), yielding matrices of size M×P, where M=19 denotes the number of EEG channels.

For Stage 2 dictionary learning, clustering was performed on $\ell_{2}$-normalized mode-based vectors using cosine dissimilarity. A hierarchical divisive clustering procedure was employed with a cluster size threshold $\tau_{\sup}=11$, minimum cluster size $n_{\min}=10$, and stopping height $h_{\mathrm{stop}}=0.2$, using complete linkage throughout. These values were determined empirically, reflecting a trade-off between marginal performance gains achievable with stricter thresholds and the substantially higher computational cost they incur. Accordingly, this configuration was adopted as a practical setting for the proposed mode-based representation. The notation and parameter settings used throughout the proposed framework are summarized in Table 2.

#### 2.6.3. Evaluation

Classification was performed with the positive class defined as AD in AD vs. CN tasks, FTD in FTD vs. CN tasks, and AD in AD vs. FTD tasks. All learning components (dictionary construction, PCA baselines, standardization, and classifier training) were restricted to training subjects in the LOSO split.

## 3. Results

This section presents the main classification results obtained using the proposed DMD-CPP framework, followed by comparative analyses with simplified DMD variants and the Ntetska2025 [21] baseline. We first report task-wise performance and margin characteristics of the proposed method and then benchmark its performance and reliability against comparable algorithms.

### 3.1. Representative Class Medoids

**Figure 3:**
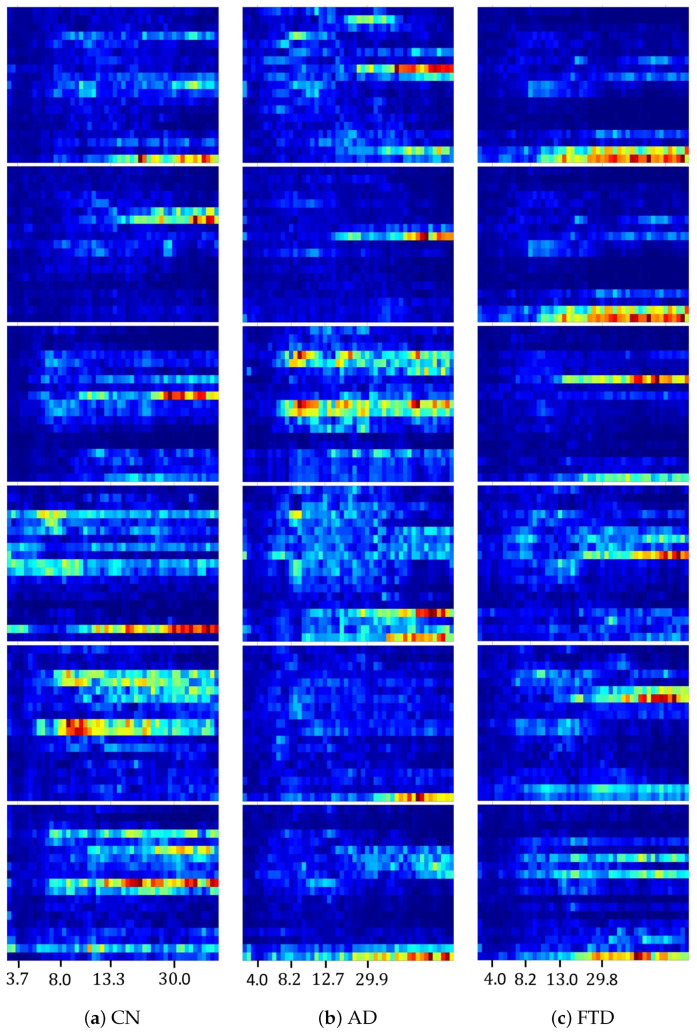
Class-specific medoid patterns learned by DMD-CPP. Shown are the top six representative medoids for each class, selected from the learned class-specific dictionaries. The ranking follows a recursive farthest-first selection, ordering medoids from those most distant to least distant in the projected feature space. Each medoid is visualized as a channel-by-frequency map derived from epoch-level projected DMD representations. Channels are ordered consistently across all columns following the fixed montage (Pz, Cz, Fz, T6, T5, T4, T3, F8, F7, O2, O1, P4, P3, C4, C3, F4, F3, Fp2, Fp1). For each class, the mean modal frequencies associated with the retained DMD modes are additionally computed and grouped into standard EEG bands, providing a band-resolved summary of dominant oscillatory content.

Figure 3 presents the top six class-specific medoids selected by the recursive farthest-point strategy used in the proposed DMD-CPP framework. Although the ranking itself is defined purely by geometric criteria in the projected feature space, the resulting ordering reveals systematic differences in the types of patterns emphasized by higher- and lower-ranked medoids. Specifically, the highest-ranked medoids tend to exhibit patterns that are most distinct from those of other classes, reflecting strong inter-class separation. These medoids often highlight localized or band-specific structures that differ markedly across classes. In contrast, as the rank decreases, medoids progressively capture patterns that are more representative of within-class variability. Lower-ranked medoids show increased similarity across samples from the same class, while their contrast with other classes becomes less pronounced.

This progression suggests that the medoid ranking implicitly spans a spectrum from discriminative prototypes, which emphasize differences relative to other classes, to more representative prototypes, which encode common internal structure within a class. Importantly, this behavior is not imposed explicitly but emerges naturally from the recursive selection procedure. At this stage, these observations are reported descriptively to characterize the structure of the learned dictionaries. Their potential neurophysiological interpretation and implications for class separability and reliability are deferred to the Discussion section.

### 3.2. Performance of the Proposed Algorithm

**Figure 4:**
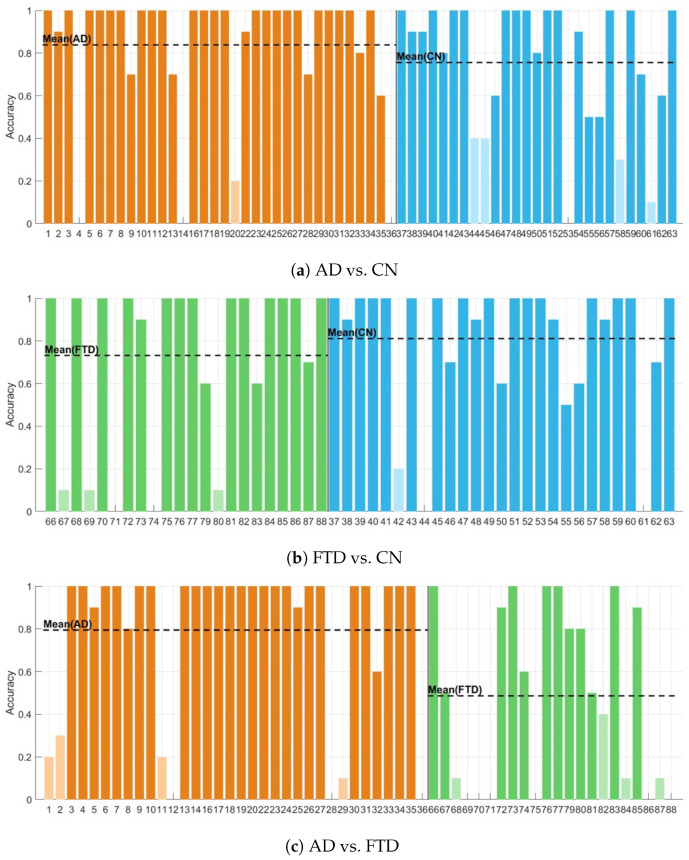
Subject-wise classification accuracy under LOSO cross-validation for three binary tasks. Each bar represents a subject’s accuracy; darker colors denote correctly classified subjects, and lighter colors indicate subjects with many misclassified epochs. Black dashed lines mark the group means.

In this subsection, we present the performance of the proposed DMD-CPP framework for each classification task, along with its subject-level variability under LOSO cross-validation. Figure 4 provides supplementary subject-wise accuracy profiles for the three binary tasks considered in this comparison (AD vs. CN, FTD vs. CN, and AD vs. FTD). Table 3 summarizes aggregated subject-level accuracies with Wilson binomial confidence intervals, whereas Table 4 reports detailed epoch-level classification metrics, including confusion matrices and class-wise precision, recall, and F1 scores.

#### 3.2.1. Metric Summary

Subject-level Summary. Subject-level results are summarized in Figure 4 and Table 3. Figure 4 illustrates the proportion of correctly classified epochs for each held-out subject under LOSO validation. Subject-wise accuracies tend to concentrate near the extremes, with many subjects achieving accuracies close to one and a smaller subset exhibiting near-zero accuracy, indicating consistent success or failure across epochs within individual subjects.

Using majority voting across epochs, subject-level accuracy reaches 0.848 for AD vs. CN and 0.833 for FTD vs. CN on decided subjects only. The corresponding 95% Wilson confidence intervals largely overlap, indicating comparable subject-level performance across the two CN-related tasks. In contrast, subject-level accuracy for AD vs. FTD drops to 0.685 with a noticeably wider confidence interval, reflecting the increased difficulty of discriminating between dementia subtypes at the subject level.

Epoch-level Summary. Epoch-level classification results are reported in Table 4. For AD vs. CN, the model achieves an epoch-level accuracy of 0.800 with balanced class-wise performance, yielding a macro-averaged F1 score of 0.798. For FTD vs. CN, epoch-level accuracy is slightly lower at 0.785, again with balanced macro-averaged metrics (macro-F1 = 0.772).

In contrast, epoch-level performance for AD vs. FTD is substantially lower, with an overall accuracy of 0.673 and noticeable asymmetry in class-wise recall and precision. This reduced performance at the epoch level is consistent with the lower subject-level accuracy observed for the same task.

Overall summary. Under strict LOSO validation, performance shows a clear task-dependent pattern. Both CN-related tasks (AD vs. CN and FTD vs. CN) achieve comparable and stable performance at the epoch and subject levels. In contrast, AD vs. FTD consistently exhibits lower accuracy and greater variability across both evaluation levels. These results indicate that classification difficulty increases substantially when discriminating between dementia subtypes compared with tasks involving controls.

#### 3.2.2. Decision-Margin Analysis

**Figure 5:**
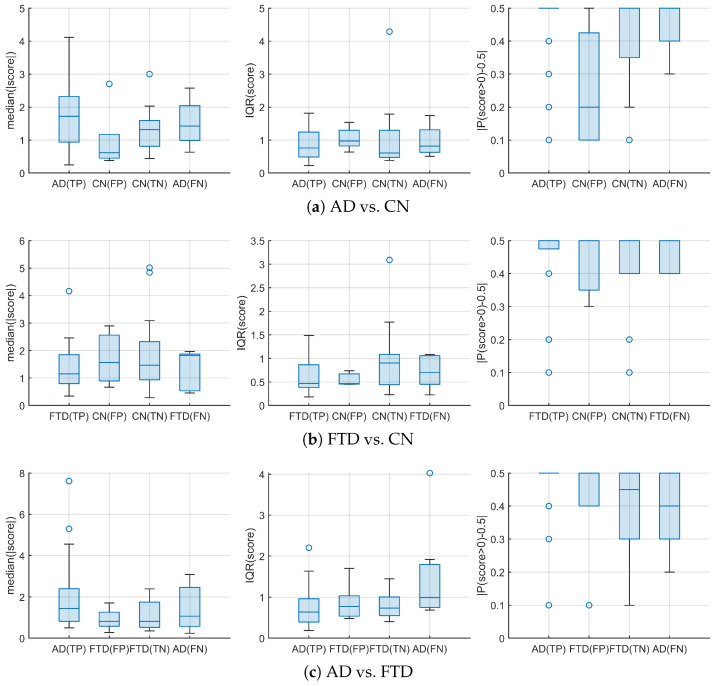
Subject-level decision–margin distributions. Boxplots depict subject-level score descriptors grouped by true → predicted outcome categories. For each task, panels show (**left**) the median absolute margin median (|m|), (**middle**) within-subject dispersion IQR (m), and (**right**) sign-consistency |P(m>0)−0.5|. Each box summarizes the distribution across subjects within the corresponding outcome group (TP, FN, TN, and FP).

Figure 5 presents the distributions of three subject-level decision-margin descriptors, as defined in Section 2.4, under LOSO validation: the median absolute margin median (|m|), the interquartile range IQR (m), and the sign-consistency index |P(m>0)−0.5|. Subjects are grouped into four outcome categories (TP, FN, TN, FP) according to majority-vote subject-level predictions. Numerical summaries of these distributions are reported in Table 5, and pairwise group-difference results based on permutation testing are shown in Table 6.

For the AD vs. CN task, margin-magnitude separation between TP and FP subjects is present but not uniform across all samples. As shown in Figure 5a, while most FP subjects exhibit relatively small median (|m|) values, one FP subject attains a comparatively large margin magnitude, which reduces the overall TP–FP separation in the distribution. This effect is reflected in the permutation-based analysis, where the TP–FP median difference for median (|m|) is positive (Δmed=1.1) but associated with a marginal permutation *p*-value ($p_{\mathrm{perm}}=0.0648$).

In contrast, the sign-consistency index |P(m>0)−0.5| exhibits a clearer TP–FP separation for the same task. As reported in Table 6, the TP–FP comparison yields a positive median difference (Δmed=0.3) with a smaller permutation *p*-value ($p_{\mathrm{perm}}=0.0417$), and a relatively large effect size (Cliff’s δ=0.63). This pattern is also visible in Figure 5a, where FP subjects show lower sign-consistency values than TP subjects, despite partial overlap in margin magnitude.

For the FTD vs. CN task, pairwise group differences are generally weak across all three descriptors. In Table 6, none of the TP–FP or TN–FN comparisons for median (|m|), IQR (m), or |P(m>0)−0.5| yields a small permutation *p*-value, and the corresponding boxplots in Figure 5b show substantial overlap between outcome groups across panels.

For the AD vs. FTD task, a TP–FP difference is observed for margin magnitude in Table 6 (Δmed=0.624, $p_{\mathrm{perm}}=0.0887$), whereas the remaining descriptors show overlapping distributions and do not exhibit a pronounced pairwise contrast. This is consistent with Figure 5c, where separation is visually limited for IQR (m) and |P(m>0)−0.5|, and overall overlap across outcome groups is substantial.

Overall, the outcome-group separation patterns vary by task: the most distinct pairwise contrasts are observed in AD vs. CN (margin magnitude and sign consistency in TP–FP), whereas tasks involving FTD show increased overlap across outcome groups and weaker pairwise differences across descriptors.

### 3.3. Benchmarking and Comparative Reliability Analysis

To contextualize the LOSO performance of the proposed DMD-CPP model, we report two complementary comparisons. First, we reference the published Ntetska2025 [21] baseline, which evaluates EO photostimulation EEG classification using 2 s PSD features and conventional machine-learning classifiers under a leave-*N*-subjects-out (LNSO) protocol. Second, we include an internal baseline that uses the same DMD-based Stage 1 representation but replaces the clustered pattern projection with PCA-based dimensionality reduction (DMD + PCA). All internal comparisons are evaluated under the same LOSO protocol as the proposed model.

#### 3.3.1. Benchmark and Metric Summary

Table 7 summarizes standard epoch-level performance metrics for the Ntetska2025 [21] baseline and the models evaluated in this study. Because the baseline employs a leave-*N*-subjects-out (LNSO) protocol, whereas our models are evaluated under strict LOSO validation, the reported results are intended to illustrate relative performance trends rather than to support a direct head-to-head comparison.

Across non-clustering baselines under EO conditions, the FTD vs. CN task shows higher accuracy than AD vs. CN. Specifically, the PSD-based baseline reports an accuracy of 76.7% for FTD vs. CN compared to 62.5% for AD vs. CN. A similar ordering is preserved by the DMD + PCA representation, which achieves accuracies of 75.2% for FTD vs. CN and 72.1% for AD vs. CN. Compared with the PSD baseline, the DMD + PCA model improves performance for AD vs. CN, while maintaining comparable accuracy for FTD vs. CN.

The proposed DMD-CPP framework yields further performance gains in both tasks. For AD vs. CN, DMD-CPP achieves an epoch-level accuracy of 80.2%, exceeding both the PSD baseline and the DMD + PCA model. For FTD vs. CN, the proposed model attains an accuracy of 77.6%, representing a modest improvement over DMD + PCA and performance comparable to the PSD baseline. As a result, the pronounced performance gap between AD vs. CN and FTD vs. CN observed in the PSD baseline is reduced under the proposed representation.

At the subject level, however, the distinction between DMD-CPP and DMD + PCA becomes less pronounced. In additional subject-wise analyses under LOSO validation, DMD-CPP achieves subject-level accuracies of 0.847 [0.735, 0.918] for AD vs. CN and 0.833 [0.720, 0.907] for FTD vs. CN, while DMD + PCA yields comparable accuracies of 0.833 [0.704, 0.913] for both tasks. The substantial overlap of the corresponding Wilson confidence intervals indicates that, based on accuracy alone, it is difficult to draw a definitive conclusion regarding the superiority of one model over the other at the subject level.

Notably, a recent study employing Riemannian connectivity features under strict LOSO validation in the EC condition reported a similar task-dependent performance pattern [14]. In that work, classification performance was higher for AD vs. CN and FTD vs. CN than for AD vs. FTD, with reported ROC–AUC values of approximately 0.72–0.89 for CN-related tasks and 0.65–0.71 for AD vs. FTD. A comparable asymmetry across tasks is also observed in the present EO-based analysis.

#### 3.3.2. Margin-Based Reliability

Beyond nominal accuracy, we evaluated subject-level reliability using margin-based statistics defined in Section 2.4. Specifically, we examined median confidence gaps derived from subject-level absolute margins, as summarized in Table 8. For each task and model, $\Delta^{\left(|m|\right)}$ denotes the median difference in |m| between correctly and incorrectly classified subjects, evaluated separately for the predicted-positive (TP vs. FP) and predicted-negative (TN vs. FN) groups. Statistical significance was assessed using permutation tests with fixed group sizes.

For the AD vs. CN task, the proposed DMD-CPP model shows larger TP–FP median confidence gaps than the DMD + PCA baseline. As reported in Table 8, the TP–FP gap reaches 1.10 under DMD-CPP ($p_{\mathrm{perm}}=0.0648$), compared with 0.52 under DMD + PCA ($p_{\mathrm{perm}}=0.2001$). In contrast, TN–FN gaps are small and not statistically distinguishable from zero for either model. These results indicate that, for AD vs. CN, margin magnitude differentiates TP and FP subjects more clearly than TN and FN subjects, with a larger separation observed under the proposed representation.

For the FTD vs. CN task, median confidence gaps are smaller and less consistent across models. Under DMD-CPP, the TP–FP gap is 0.52 ($p_{\mathrm{perm}}=0.6324$), while the TN–FN gap is −0.17 ($p_{\mathrm{perm}}=0.8127$). Comparable values are observed for DMD + PCA, and none of the pairwise contrasts reach statistical significance. This pattern indicates substantial overlap in subject-level margin magnitudes between correctly and incorrectly classified subjects for this task.

For the AD vs. FTD task, both models exhibit moderate TP–FP gaps of similar magnitude (0.62 for DMD-CPP and 0.62 for DMD + PCA), with permutation *p*-values of 0.0887 and 0.0922, respectively. In contrast, TN–FN gaps remain small and non-significant for both representations. Overall, the magnitude and statistical strength of margin-based gaps for AD vs. FTD are weaker and less consistent than those observed for AD vs. CN.

Across tasks, margin-based reliability patterns vary substantially. The largest and most consistent median confidence gaps are observed for AD vs. CN in the TP–FP comparison, whereas tasks involving FTD exhibit reduced separation and increased overlap in subject-level margin magnitudes.

## 4. Discussion

This section summarizes the main findings of the proposed DMD-CPP framework and discusses their methodological implications under EO stimulation. We first highlight task-dependent performance characteristics observed across AD vs. CN, FTD vs. CN, and AD vs. FTD classifications. We then describe the properties of the class-specific medoid patterns learned through clustering and examine how these representations shape decision-margin behavior. Finally, we discuss the limitations of the current study and relate our findings to prior EEG-based dementia research.

### 4.1. Summary of the Analytical Approach

This study investigates whether stimulus-interval EO EEG can provide diagnostically meaningful information for neurodegenerative diseases when represented through data-driven dynamic patterns derived from DMD. Using a publicly available OpenNeuro dataset, the analysis was restricted to visually driven stimulus periods, from which a uniform set of ten non-overlapping 20 s epochs per subject was constructed to ensure balanced subject-level evaluation. Extended DMD with temporal stacking was applied to each 2 s segment to extract channel-level modes that capture transient spatiotemporal dynamics beyond stationary spectral descriptors. The extracted mode-based summaries were assembled into fixed-length representations and organized using cosine-based hierarchical divisive clustering. This procedure yielded class-specific medoid dictionaries that encode representative dynamic patterns learned directly from the training data. Each epoch was then projected onto these dictionaries via cosine similarity, forming the DMD-CPP feature representation. Linear SVMs were trained under a strict LOSO protocol to ensure subject-wise generalization without information leakage.

To contextualize the proposed approach, two simplified DMD-based pipelines were evaluated: one that retained clustering-based projection on direct mode summaries and another that replaced clustering with PCA-based dimensionality reduction. In addition, the results were compared with a previously reported baseline using classical machine-learning models on short-window PSD features. Together, these comparisons enable the examination of the role of pattern-level clustering, projection, and margin-based reliability independently of nominal accuracy.

### 4.2. Headline Findings

Across the three binary classification tasks, the proposed DMD-CPP framework exhibited consistent yet strongly task-dependent behavior under EO photostimulation, highlighting limitations of conventional interpretations based solely on signal preservation or spectral clarity.

Under EC conditions, prior studies have consistently reported that AD vs. CN classification achieves the highest performance, supported by relatively stable and disease-specific low-frequency connectivity patterns, while FTD vs. CN shows moderately reduced accuracy due to greater heterogeneity, and AD vs. FTD remains the most challenging task owing to substantial overlap between dementia subtypes [11,12,14,27]. This EC-based performance hierarchy has often served as an implicit reference for interpreting EEG-based dementia results.

Under EO conditions, however, this hierarchy is typically disrupted. Alzheimer-related oscillatory activity is more severely attenuated and unstable during visual stimulation, leading many EO EEG studies to report poorer performance for AD vs. CN than for FTD vs. CN [21,28]. In contrast to this prevailing view, the proposed method yielded a larger relative improvement for AD vs. CN than for FTD vs. CN, elevating AD vs. CN performance to a level comparable with FTD vs. CN. Importantly, this improvement was observed although Alzheimer’s disease is known to exhibit pronounced disruption of visually driven neural dynamics under EO conditions, where conventional physiological signatures are often considered difficult to capture. By comparison, the FTD vs. CN task showed only moderate improvement, with residual variability and weaker separability. This behavior is consistent with the known heterogeneity of FTD and the partial overlap between FTD and control EEG dynamics, which limits the formation of sharply separable representations under clustering-based learning.

Taken together, these findings indicate that the improved margin behavior observed in AD-related tasks cannot be straightforwardly explained by preserved or cleaner EEG dynamics. Rather, they point to the need for an alternative analytical perspective that goes beyond conventional spectral or signal-quality-based accounts of EO EEG, motivating a reconsideration of how disease-related information is expressed and captured under stimulus-driven conditions.

### 4.3. Interpreting Medoid Patterns

Before discussing the margin asymmetries induced by DMD-CPP, it is necessary to clarify how the learned medoid patterns should be understood. Figure 3 visualizes class-specific medoids learned by the proposed framework, and the following description should be read as a characterization of observed pattern structure rather than as a definitive physiological interpretation. In this study, medoids are not regarded as biologically prototypical or canonical EEG signatures. Instead, they represent recurrent configurations of oscillatory degradation, reflecting how spatiotemporal organization is altered or weakened under disease-specific conditions during EO stimulation.

Across all three groups (CN, AD, and FTD), a shared characteristic is that higher-frequency components associated with visually driven activity remain relatively localized, whereas lower-frequency structure becomes attenuated, fragmented, or unstable. The primary distinction across groups lies not in the presence of a fixed spectral template but in the manner by which low-frequency information diminishes and interacts with higher-frequency activity.

For both AD and FTD, low-frequency modes are generally reduced in strength relative to CN, and many medoids exhibit limited low-frequency continuity. In the upper-ranked medoids, some patterns from AD and FTD appear visually similar to CN, reflecting subject-specific or idiosyncratic realizations that may contribute to classification ambiguity at the subject level. Such patterns are consistent with the observed difficulty of subtype discrimination and the presence of atypical samples in the AD vs. FTD task. As medoid rank decreases toward more general representatives, clearer group-dependent tendencies emerge. In AD, low-frequency structure often appears increasingly blurred or erased, suggesting a progressive collapse of organized low-frequency dynamics. In contrast, FTD medoids tend to show a more uniform weakening of low-frequency components without converging toward a single dominant degradation pattern. Although these degradation modes differ in appearance, their internal similarity allows clustering to identify representative medoids that summarize common modes of breakdown.

CN medoids exhibit a different behavior. Across ranks, patterns remain heterogeneous and subject-specific, yet low-frequency components retain comparatively richer structure and continuity. Rather than converging toward a failure mode, CN medoids reflect normal inter-individual variability and preserved low-frequency organization supporting transitions to higher-frequency activity.

Taken together, these observations indicate that DMD-CPP medoids do not encode fixed disease templates but instead summarize recurrent ways in which oscillatory structure weakens under EO stimulation. This descriptive characterization provides the basis for understanding subsequent task-dependent margin behavior, which is examined in the following subsection.

### 4.4. Methodological Interpretation: Clustering-Based Basis Learning Under EO Conditions

Compared with the EC state, the EO photic condition is generally considered more challenging for differentiating AD from healthy controls when using conventional spectral representations [21,39]. Under EO stimulation, posterior alpha rhythms are attenuated and less regular, and visually driven responses in AD often exhibit reduced or unstable entrainment, characterized by diminished harmonic power and weakened interhemispheric coherence relative to controls [19,40]. Such instability of steady-state visual responses has been interpreted as a breakdown of large-scale synchronization and impaired neural coupling in AD, thereby limiting the discriminative power of short-window spectral features.

As a consequence, features derived directly from short-window spectral power, covariance, or PSD estimates tend to show increased variability and weaker class separation under EO conditions than under EC resting states [11]. This degradation reflects not only alpha suppression but also the fragmented and stimulus-dependent nature of EO spectral patterns, which vary substantially across epochs and subjects [20]. Within this context, the clustering component of the proposed framework plays a central methodological role. Rather than assuming the existence of stable or prototypical disease-specific spectral templates, high-dimensional DMD-based representations are aggregated through hierarchical divisive clustering to form a compact dictionary of basis exemplars (medoids). These medoids summarize recurrent modes of spatiotemporal organization present in the training data, including characteristic patterns of oscillatory degradation. Each new epoch is subsequently projected onto these learned bases using cosine similarity, yielding normalized coordinates that quantify structural alignment with class-specific modes of organization or disorganization.

This clustering–projection mechanism is particularly suited to EO conditions, where AD-related responses tend to appear fragmented and inconsistent at the single-epoch level. By emphasizing shared geometric structure across epochs, clustering reduces the influence of transient amplitude fluctuations and inter-subject variability, while projection expresses each sample relative to group-level reference modes. Importantly, these reference modes are not interpreted as biologically preserved signatures but as reproducible structural configurations learned from data.

The larger gains observed for AD vs. CN—relative to the more modest improvements for FTD vs. CN—are therefore consistent with differences in how low-frequency degradation patterns manifest across diseases. In AD, EO-related disorganization appears to recur in a sufficiently structured manner across subjects and epochs to be consolidated through clustering. In contrast, greater heterogeneity and fewer recurring degradation modes in FTD limit the degree to which clustering can stabilize the representation. Overall, these results suggest that clustering-based basis learning can recover discriminative structure from EO EEG by organizing recurring modes of oscillatory disruption that are otherwise treated as noise in conventional analyses.

### 4.5. Interpretation of Asymmetric Margin Structure

An important observation of this study is the asymmetric margin structure induced by the proposed DMD-CPP framework under EO photostimulation. Specifically, margin separability differs across tasks and classes, with AD-related decisions exhibiting more pronounced and controllable margin behavior than those involving CN or FTD. This asymmetry is not fully explained by nominal classification accuracy alone but reflects how class-dependent structure is organized through clustering-based basis learning.

Clustering-based representations are not inherently robust when classes lack recurrent or consolidatable structure. When patterns are highly heterogeneous or sample sizes are limited, clustering may preserve idiosyncratic realizations rather than forming stable prototypes. From this perspective, the relatively weak margin structure observed for FTD vs. CN is consistent with the known clinical and neurophysiological heterogeneity of FTD, whose EEG alterations are less stereotyped and more regionally variable than those of AD [41]. Combined with a smaller sample size, this heterogeneity constrains the formation of stable cluster anchors and limits margin-based confidence stratification.

By contrast, the behavior observed for AD suggests a different structural regime. Although AD does not exhibit a stable EEG pattern in the traditional sense, it is associated with relatively consistent breakdowns of large-scale oscillatory coupling, particularly in posterior and long-range networks [42,43]. Under EO conditions, this breakdown has been reported as fragmented visual entrainment, reduced phase coherence, and impaired interregional synchronization [44,45], while such responses appear irregular at the single-epoch level, their structural characteristics may recur across epochs and subjects. Both DMD + PCA and DMD-CPP capture this separability in the AD vs. FTD task, as reflected by significant margin differences under both representations. This suggests that low-frequency degradation patterns in AD and FTD, although both pathological, remain distinguishable at a structural level. The additional clustering stage in DMD-CPP appears to further organize these recurring AD-related configurations into more stable reference modes.

As a result, control epochs rarely align strongly with AD-related bases. When CN epochs are misclassified as AD, their similarity to AD anchors remains low, leading to systematically smaller decision margins. From a reliability standpoint, this implies that false-positive AD decisions tend to be associated with low confidence, making them amenable to margin-based screening or rejection. Importantly, this behavior should not be interpreted as evidence of preserved biological signatures. Rather, it indicates that the mode of oscillatory breakdown in AD may itself constitute a reproducible structural pattern that clustering-based representations can exploit under strict LOSO validation.

Conversely, AD epochs misclassified as CN exhibit more variable margins, consistent with the diffuse and heterogeneous structure of the CN feature space under EO conditions. Normal responses span a broad range of stimulus-dependent and subject-specific dynamics, limiting the formation of a strong central attractor and constraining margin separation near the decision boundary.

Overall, these results indicate that the primary contribution of DMD-CPP lies not in uniformly increasing accuracy but in reshaping decision geometry in a task- and class-dependent manner. Clustering-based basis learning is most effective when pathological processes give rise to recurrent structural configurations, as observed for AD under EO stimulation. When such recurrence is limited, as in FTD, the benefits of clustering diminish. This behavior underscores both the potential and the limitations of clustering-driven representations under strict LOSO evaluation.

### 4.6. Limitations

Several limitations of this study should be acknowledged.

First, EEG signals exhibit substantial inter- and intra-subject variability arising from vigilance fluctuations, medication effects, and recording-session factors. Under the LOSO protocol, a small subset of subjects displayed margin patterns that were inconsistent with their clinical labels. Such cases are likely attributable to individual neurophysiological idiosyncrasies rather than to systematic model failure, a well-known challenge in subject-level EEG classification [46,47,48].

Second, although the DMD-CPP framework stabilizes heterogeneous EO responses through clustering-based basis learning, its effectiveness depends on the availability of sufficiently recurrent class-specific structures in the training data. In particular, the smaller sample size and higher physiological heterogeneity of the FTD cohort may have limited the formation of stable and representative prototypes, thereby constraining margin-based separability for FTD-related tasks.

Relatedly, inspection of the learned medoid patterns (Figure 3) reveals clear class-dependent differences in the spectral breadth and density of DMD modes across frequency bands. AD, FTD, and CN exhibit distinct distributions in terms of how many modes are retained within low- and high-frequency ranges, reflecting different forms of spectral organization or degradation. However, the current framework does not explicitly encode or normalize these band-wise mode-count differences. As a result, subjects whose overall spectral geometry deviates from the dominant class-specific mode distribution—despite sharing similar clinical characteristics—may be misclassified. This limitation suggests that some errors arise not from a lack of discriminative structure but from an incomplete utilization of band-dependent mode complexity in the decision process.

Third, DMD is computationally demanding for long continuous recordings. To ensure tractability, our analysis relied on short (2-s) segments aggregated into 20 s epochs, which may underrepresent slower coupling dynamics or long-range coordination processes [30]. Future work incorporating multi-scale or adaptive windowing strategies may help capture such effects more comprehensively.

Finally, the present study focused on DMD-derived representations combined with cosine-based clustering and prototype projection. We did not investigate whether the observed margin asymmetries are specific to DMD-based features or would also emerge when similar clustering–projection schemes are applied to alternative EEG representations, such as spectral, covariance-based, or data-driven latent features. Consequently, it remains an open question whether the reliability gains observed here reflect a unique advantage of the DMD-CPP formulation or a more general property of prototype-based feature learning in high-dimensional EEG spaces.

### 4.7. Positioning with Respect to Prior EEG-Based Dementia Studies

Most prior EEG-based dementia studies have focused on EC conditions, where relatively stable posterior alpha rhythms enable reliable characterization using spectral power, covariance structure, or deep learning-based representations. Under such settings, averaging-based or stationary descriptors are often effective, and extensive comparative evaluations have been reported. However, these assumptions do not readily extend to EO stimulation, where alpha suppression, stimulus-dependent nonstationarity, and fragmented oscillatory responses substantially reduce the robustness of conventional spectral features [21,39].

To date, EEG-based dementia classification studies conducted explicitly under EO photostimulation remain extremely limited. For the OpenNeuro ds006036 dataset analyzed in this work, prior EO-based classification studies are essentially restricted to two reports from the data-providing group: an earlier study based on short-window PSD features combined with conventional machine-learning classifiers [21], and a more recent CNN-based approach [28]. While these studies demonstrate the feasibility of EO-based discrimination, they primarily report nominal classification performance and do not provide analyses of subject-level reliability, decision confidence, or margin-based error structure under strict LOSO validation. As a result, constructing a direct quantitative comparison across EO-based approaches remains challenging.

In this context, one of the central contributions of the present study is the analysis of decision reliability through subject-level margin statistics under LOSO validation. To the best of our knowledge, margin-based reliability analyses—particularly those examining how decision confidence differs between correct and incorrect subject-level outcomes—have rarely been reported in EEG-based dementia studies, under either EO or EC conditions. Accordingly, there are currently no established benchmarks against which the confidence structure revealed by the proposed framework can be directly compared.

Rather than positioning the proposed DMD-CPP framework as an accuracy-driven alternative to existing methods, we emphasize its role as a reliability-oriented analytical approach for challenging EO EEG data. By organizing DMD-derived representations through clustering-based basis learning and projection, the method provides a structured feature space in which subject-level decision margins can be examined and interpreted. In particular, the framework enables systematic investigation of when margin-based confidence is informative and when it breaks down, as observed in the contrasting behaviors between AD vs. CN and FTD-related tasks. In this sense, the primary contribution of the present work lies not in claiming superiority over scarce EO-based baselines but in offering a principled framework for characterizing the reliability and limitations of EO-based dementia classification, especially for Alzheimer’s disease.

### 4.8. Future Directions

Building on the present findings, future work will shift the focus from detecting prototypical EEG patterns to explicitly characterizing pathological breakdown of neural dynamics. In EO conditions, Alzheimer’s disease is not primarily associated with the emergence of a stable alternative pattern but rather with the progressive collapse, fragmentation, or loss of low-frequency organization that is typically preserved in healthy subjects. This property fundamentally challenges conventional learning-based classifiers, which are designed to identify recurring and coherent templates.

Methodologically, we aim to develop models that treat such breakdown phenomena themselves as informative structures. Rather than forcing pathological EEG to match a representative prototype, future frameworks will quantify the degree, persistence, and variability of dynamical collapse across time and frequency. In this direction, we will investigate multi-scale and incremental variants of DMD to capture slow drifts, cross-epoch instability, and long-range temporal dependencies without incurring prohibitive computational cost.

Beyond DMD, the proposed reliability-oriented perspective will be extended to a broader class of nonlinear dynamical descriptors, including Riemannian covariance geometry, entropy-based measures, empirical mode decomposition (EMD), and related state-space representations. The goal is not only to improve nominal classification accuracy but to construct unified measures that explicitly distinguish between stable physiological organization and pathological disintegration. Such measures are expected to provide meaningful explanations for both correct and failed classifications, thereby supporting confidence-aware and interpretable decision-making.

## 5. Conclusions

This study proposed a DMD-CPP framework for analyzing photic-stimulation EEG under EO conditions. By combining mode-based representations with clustering-driven basis learning, the framework constructs a structured and interpretable feature space that is robust to the substantial inter- and intra-subject variability inherent in EO EEG.

Importantly, the primary contribution of this work does not lie solely in algorithmic performance gains but in the insight that Alzheimer’s disease—despite lacking a stable or stereotyped EEG signature—may exhibit a consistent structure of breakdown that is reproducible across subjects. Although individual EO EEG realizations from AD patients differ markedly, the manner in which low-frequency organization degrades and high-frequency components dominate appears to follow a coherent geometric pattern. The DMD-CPP framework captures this phenomenon by learning prototype representations that encode how neural dynamics fail, rather than what a canonical disease pattern looks like.

Unlike conventional spectral or covariance-based approaches, which often treat such fragmented EO responses as noise, the proposed clustering–projection mechanism aggregates these heterogeneous realizations into a set of representative medoid bases. Each epoch is then expressed relative to these bases, enabling reliable subject-level discrimination under strict LOSO evaluation—even when test subjects exhibit patterns not seen during training.

Empirically, this property manifests as strong margin-based reliability for AD-related discrimination. In particular, samples that are ambiguous or atypical (e.g., CN→AD errors) are systematically assigned low decision margins, indicating conservative and controllable behavior rather than overconfident misclassification. Crucially, this margin separation emerges even though LOSO evaluation ensures that test subjects’ EEG patterns differ from those used to construct the medoids. This suggests that the learned representations capture a generalizable failure structure of AD rather than subject-specific or idiosyncratic features.

While several limitations remain—including the computational cost of DMD and the absence of exhaustive comparisons with alternative EEG representations—the present findings demonstrate that prototype-based learning over DMD-derived dynamics offers a robust and interpretable pathway for EEG analysis under challenging EO conditions.

## Figures and Tables

**Table 1:** Summary of visual stimulus onset and offset times (in seconds) and the corresponding stimulus durations for each subject in the AD, CN, and FTD groups.

Alzheimer’s Disease (AD)			Normal Control (CN)			Frontotemporal Dementia (FTD)		
ID	[Onset, Offset]	Duration	ID	[Onset, Offset]	Duration	ID	[Onset, Offset]	Duration
1	[3.80, 67.46]	63.66	37	[6.48, 76.47]	69.99	66	[1.07, 71.40]	70.33
2	[16.89, 105.14]	88.25	38	[14.82, 79.02]	64.20	67	[16.01, 98.93]	82.92
3	[0.03, 43.93]	43.90	39	[14.94, 162.02]	147.08	68	[0.30, 76.82]	76.52
4	[15.35, 103.34]	88.00	40	[0.52, 59.34]	58.81	69	[14.25, 96.26]	82.01
5	[5.37, 83.76]	78.39	41	[19.09, 107.59]	88.49	70	[7.11, 97.10]	89.99
6	[14.37, 118.39]	104.03	42	[16.49, 106.49]	89.99	71	[3.88, 86.16]	82.28
7	[17.39, 104.14]	86.75	43	[7.25, 156.29]	149.04	72	[0.95, 89.94]	89.00
8	[15.57, 102.07]	86.51	44	[9.52, 95.68]	86.15	73	[18.57, 102.07]	83.49
9	[21.89, 104.33]	82.43	45	[1.51, 111.43]	109.92	74	[4.23, 91.69]	87.45
10	[15.08, 93.16]	78.08	46	[14.29, 104.28]	89.99	75	[12.63, 107.28]	94.64
11	[13.99, 102.49]	88.50	47	[14.33, 103.83]	89.49	76	[10.85, 87.56]	76.70
12	[2.99, 84.01]	81.02	48	[4.57, 92.76]	88.19	77	[8.49, 98.48]	89.99
13	[17.18, 105.55]	88.37	49	[27.09, 113.70]	86.60	78	[8.11, 37.61]	29.50
14	[10.05, 97.89]	87.84	50	[9.34, 92.11]	82.76	79	[14.16, 81.65]	67.50
15	[0.45, 28.34]	27.89	51	[12.25, 100.75]	88.50	80	[8.06, 71.81]	63.76
16	[5.05, 95.04]	89.99	52	[2.08, 91.07]	88.99	81	[21.40, 85.26]	63.86
17	[19.54, 106.29]	86.75	53	[9.55, 94.01]	84.46	82	[3.81, 69.06]	65.25
18	[24.73, 114.72]	89.99	54	[3.83, 147.79]	143.96	83	[1.40, 65.90]	64.51
19	[0.68, 90.67]	89.99	55	[15.39, 91.90]	76.51	84	[2.29, 67.62]	65.33
20	[0.95, 89.95]	89.00	56	[17.39, 106.14]	88.75	85	[27.02, 92.02]	65.01
21	[18.10, 55.15]	37.04	57	[0.06, 68.21]	68.14	86	[9.73, 73.93]	64.20
22	[4.05, 89.44]	85.38	58	[0.05, 52.68]	52.63	87	[5.51, 71.77]	66.25
23	[2.92, 64.13]	61.21	59	[0.45, 54.49]	54.04	88	[56.91, 122.91]	66.00
24	[6.33, 69.09]	62.76	60	[0.74, 126.97]	126.23			
25	[3.81, 73.80]	69.99	61	[6.05, 72.80]	66.75			
26	[12.33, 80.32]	68.00	62	[16.58, 86.07]	69.49			
27	[4.97, 92.47]	87.50	63	[2.74, 69.16]	66.42			
28	[3.89, 55.05]	51.16	64	[0.03, 23.48]	23.45			
29	[26.26, 90.77]	64.52	65	[0.03, 18.26]	18.23			
30	[1.59, 71.08]	69.49						
31	[6.77, 74.77]	68.00						
32	[2.79, 67.79]	65.00						
33	[25.86, 94.77]	68.90						
34	[15.22, 74.24]	59.02						
35	[18.33, 98.75]	80.42						
36	[5.32, 74.32]	69.00						

**Table 2:** Notation used in the proposed framework. Symbols include design parameters introduced by the method.

Symbol	Meaning
N=1000	Number of samples per segment (2 s at 500 Hz)
T=10	Number of segments per epoch
S=48	DMD stack size (see Equation (A1))
R=100	DMD truncation rank (see Equation (A1))
P=50	Mode-axis resolution for epoch descriptors (see Equation (3))
$\tau_{\sup}=11$	Cluster size threshold
$n_{\min}=10$	Minimum cluster size
$h_{\mathrm{stop}}=0.2$	Divisive clustering stopping height

**Table 3:** Subject-level performance under LOSO validation, including rejection. Accuracy is computed on decided subjects only. Wilson’s binomial confidence intervals are reported for conditional accuracy.

Task	Accuracy	95% Wilson CI	Reject Rate	Confusion
AD vs. CN	0.848	[0.735, 0.918]	3.3%	TP = 30, FN = 4, FP = 5, TN = 20
FTD vs. CN	0.833	[0.704, 0.913]	2.0%	TP = 17, FN = 5, FP = 3, TN = 23
AD vs. FTD	0.685	[0.553, 0.793]	3.6%	TP = 27, FN = 7, FP = 10, TN = 10

**Table 4:** Epoch-level Summary. Confusion matrices (left) and class-wise metrics (right). Macro-averaged values and overall accuracy are shown at the bottom of each block.

Task		Confusion Matrix		Class-Wise Metrics		
		Pred. CN/FTD	Pred. AD/FTD	Precision	Recall	F1
AD vs. CN	True CN	204	66	0.788	0.756	0.771
	True AD	55	285	0.812	0.838	0.825
	Macro/Acc	Acc = 0.802		0.800	0.797	0.798
FTD vs. CN	True CN	219	51	0.788	0.811	0.799
	True FTD	59	161	0.759	0.732	0.745
	Macro/Acc	Acc = 0.776		0.734	0.772	0.772
AD vs. FTD	True FTD	107	113	0.605	0.486	0.539
	True AD	70	270	0.705	0.794	0.747
	Macro/Acc	Acc = 0.673		0.655	0.640	0.643

**Table 5:** Subject-level score distribution statistics under LOSO validation. Each subject is assigned to one of four outcome groups (positive correct, positive miss, negative correct, negative miss) based on majority-vote prediction. For each descriptor, the median is shown on the first line, and the interquartile range (25th–75th percentile) on the second line.

Task	Group	*N*	Median (|m|)	IQR (m)	|P(m>0)−0.5|
AD vs. CN	AD-correct (TP)	30	1.721 [0.939, 2.321]	0.757 [0.487, 1.244]	0.500 [0.500, 0.500]
	CN-miss (FP)	5	0.619 [0.449, 1.168]	0.973 [0.821, 1.295]	0.200 [0.100, 0.425]
	CN-correct (TN)	20	1.318 [0.811, 1.589]	0.608 [0.476, 1.299]	0.500 [0.350, 0.500]
	AD-miss (FN)	4	1.423 [0.985, 2.042]	0.815 [0.627, 1.312]	0.500 [0.400, 0.500]
FTD vs. CN	FTD-correct (TP)	17	1.150 [0.796, 1.851]	0.468 [0.384, 0.866]	0.500 [0.475, 0.500]
	CN-miss (FP)	5	1.563 [0.890, 2.564]	0.465 [0.455, 0.669]	0.500 [0.350, 0.500]
	CN-correct (TN)	23	1.464 [0.932, 2.322]	0.902 [0.441, 1.087]	0.500 [0.400, 0.500]
	FTD-miss (FN)	4	1.826 [0.530, 1.875]	0.703 [0.450, 1.058]	0.400 [0.400, 0.500]
AD vs. FTD	AD-correct (TP)	27	1.442 [0.818, 2.397]	0.641 [0.395, 0.964]	0.500 [0.500, 0.500]
	FTD-miss (FP)	10	0.818 [0.577, 1.263]	0.773 [0.539, 1.037]	0.500 [0.400, 0.500]
	FTD-correct (TN)	10	0.820 [0.527, 1.751]	0.735 [0.551, 1.006]	0.450 [0.300, 0.500]
	AD-miss (FN)	7	1.070 [0.573, 2.467]	0.993 [0.751, 1.791]	0.400 [0.300, 0.500]

**Table 6:** Permutation-based group-difference analysis of subject-level margin descriptors. For each task, we compare the predicted-positive group (TP vs. FP) and the predicted-negative group (TN vs. FN) using the median difference, Δmed, defined as the difference between the group-wise median values of a given margin descriptor, together with bootstrap confidence intervals (CI), permutation *p*-values ($p_{\mathrm{perm}}$; 20,000 permutations with fixed group sizes), and Cliff’s δ effect size with CI.

Task	Descriptor	Compare	Δmed [CI]	$p_{\mathrm{perm}}$	Cliff’s δ [CI]	$n_{1}$ vs. $n_{2}$
AD vs. CN	median (|m|)	(TP–FP)	1.1 [−0.989, 1.49]	0.0648	0.44 [−0.213, 0.907]	30 vs. 5
		(TN–FN)	−0.106 [−1.27, 0.684]	0.75	−0.23 [−0.8, 0.4]	20 vs. 4
	IQR (m)	(TP–FP)	−0.215 [−0.777, 0.128]	0.355	−0.35 [−0.693, 0.0533]	30 vs. 5
		(TN–FN)	−0.207 [−1.15, 0.308]	0.497	−0.20 [−0.7, 0.35]	20 vs. 4
	|P (m>0)−0.5|	(TP–FP)	0.3 [0, 0.4]	0.0417	0.63 [0.16, 0.96]	30 vs. 5
		(TN–FN)	0 [−0.1, 0.2]	1.0	−0.15 [−0.55, 0.375]	20 vs. 4
FTD vs. CN	median (|m|)	(TP–FP)	−0.412 [−2.01, 1.01]	0.632	−0.22 [−0.882, 0.529]	17 vs. 3
		(TN–FN)	−0.361 [−0.888, 1.5]	0.813	0.25 [−0.304, 0.739]	23 vs. 5
	IQR (m)	(TP–FP)	0.0033 [−0.311, 0.357]	1.0	−0.10 [−0.569, 0.412]	17 vs. 3
		(TN–FN)	0.198 [−0.474, 0.676]	1.0	0.10 [−0.478, 0.652]	23 vs. 5
	|P (m>0)−0.5|	(TP–FP)	0 [0, 0.2]	1.0	0.06 [−0.353, 0.647]	17 vs. 3
		(TN–FN)	0.1 [0, 0.1]	0.290	0.15 [−0.287, 0.6]	23 vs. 5
AD vs. FTD	median (|m|)	(TP–FP)	0.624 [0.0131, 1.29]	0.0887	0.52 [0.17, 0.807]	27 vs. 10
		(TN–FN)	−0.25 [−2.18, 1.09]	0.754	−0.0857 [−0.657, 0.543]	10 vs. 7
	IQR (m)	(TP–FP)	−0.131 [−0.478, 0.286]	0.636	−0.19 [−0.556, 0.193]	27 vs. 10
		(TN–FN)	−0.258 [−1.37, 0.159]	0.227	−0.51 [−0.914, 0.0286]	10 vs. 7
	|P (m>0)−0.5|	(TP–FP)	0 [0, 0.1]	1.0	0.24 [−0.0889, 0.574]	27 vs. 10
		(TN–FN)	0.05 [−0.15, 0.2]	0.983	0.1 [−0.429, 0.629]	10 vs. 7

**Table 7:** Performance comparison across methods. Baseline ([21]) uses LNSO; current models use LOSO. Values are for trend comparison only.

Task	Method	Acc.	Prec.	Rec.	F1
AD vs. CN	Baseline (PSD + SVM, LNSO)	62.5	57.6	79.6	66.8
	DMD + PCA (Linear-SVM)	72.1	71.8	71.9	71.8
	Proposed: DMD-CPP (Linear SVM)	80.2	79.9	79.7	79.8
FTD vs. CN	Baseline (PSD + LightGBM, LNSO)	76.7	76.7	76.1	76.3
	DMD + PCA (Linear-SVM)	75.2	75.3	75.0	75.6
	Proposed: DMD-CPP (Linear SVM)	77.6	77.4	77.2	77.2

**Table 8:** Permutation-based evaluation of median confidence gaps under LOSO validation. For each task and model, $\Delta^{\left(|m|\right)}$ denotes the difference between the medians of |m| for correctly and incorrectly classified subjects (TP vs. FP and TN vs. FN). Statistical significance is assessed using permutation tests with fixed group sizes.

Task	Model	$\Delta_{\mathrm{TP}-\mathrm{FP}}^{\left(|m|\right)}$	$p_{\mathrm{perm}}$	$\Delta_{\mathrm{TN}-\mathrm{FN}}^{\left(|m|\right)}$	$p_{\mathrm{perm}}$
AD vs. CN	Proposed	1.10	0.0648	−0.11	0.75
	DMD + PCA	0.52	0.2001	−0.17	0.7993
FTD vs. CN	Proposed	0.52	0.6324	−0.17	0.8127
	DMD + PCA	0.36	0.7130	0.34	0.5077
AD vs. FTD	Proposed	0.62	0.0887	−0.25	0.7542
	DMD + PCA	0.62	0.0922	−0.08	0.8508

## Data Availability

The source code is available at https://www.mathworks.com/matlabcentral/fileexchange/183208-dmd-cpp (accessed on 5 February 2026), the code named DMDCPP.m, and the original data presented in the study are openly available in the OpenNeuro repository at https://openneuro.org/datasets/ds006036/versions/1.0.4 (accessed on 5 February 2026).

## Abbreviations

The following abbreviations are used in this manuscript:

DMD	Dynamic Mode Decomposition
CPP	Clustered Pattern Projection
LOSO	Leave-One-Subject-Out
LNSO	Leave-*N*-Subjects-Out
AD	Alzheimer’s Disease
CN	Healthy Control
FTD	Frontotemporal Dementia
EO	Eyes-Open
EC	Eyes-Closed
MRI	Magnetic Resonance Imaging
fMRI	functional Magnetic Resonance Imaging
PET	Positron Emission Tomography
CI	Confidence Interval
SD	Standard Deviation
SVD	Singular Value Decomposition
SVM	Support Vector Machine
PCA	Principal Component Analysis
PSD	Power Spectral Density

## Appendix A. Mathematical Details

### Appendix A.1. Dynamic Mode Decomposition

DMD provides a data-driven framework for decomposing multichannel EEG into coherent spatio–temporal patterns. Formally, given a multivariate signal

$$X={\left\{x\left(t_{k}\right)\right\}}_{k=1}^{N},\;x\left(t_{k}\right)\in R^{M},$$

we construct a stacked Hankel matrix with stack size *S*,

$$Y^{\left(S\right)}\in R^{MS\times N_{S}},\;N_{S}=N-S+1,$$

where each column concatenates *S* consecutive *M*-dimensional samples. This augmentation, often referred to as Extended DMD, expands the state dimension from *M* to MS and improves the spectral resolution of the decomposition.

The DMD algorithm approximates a linear mapping A such that

$$Y_{2}\approx A\;Y_{1},$$

where $Y_{1},Y_{2}\in R^{MS\times (N_{S}-1)}$ are time-shifted blocks of $Y^{\left(S\right)}$. A low-rank surrogate $\tilde{A}$ of dimension *R* is obtained via truncated SVD, leading to the eigendecomposition

(A1) $$\tilde{A}\Phi =\Phi \Lambda .$$

Here, $\Phi \in C^{MS\times R}$ contains the DMD modes, $\Lambda =\mathrm{diag}(\lambda_{1},\ldots ,\lambda_{R})$ contains the eigenvalues, and the mode amplitudes are encoded in a vector c. Each eigenvalue $\lambda_{j}$ defines an oscillatory component with frequency

(A2) ωj=Im(logλj)2πΔt.

In practice, the triplet (Φ,λ,c) captures spatial patterns, temporal evolution, and mode amplitudes, respectively.

In this study, we employed a stack size of S=48 (corresponding to 96 ms at a sampling rate of 500 Hz) and a truncation rank of R=100. The stacking depth was chosen such that the augmented state dimension exceeds the original channel dimension by approximately a factor of two, following common practice in time-delay and Hankel DMD formulations, where sufficiently large delay embeddings are required to stabilize spectral estimation and capture coherent oscillatory dynamics [49]. The truncation rank was selected to balance spectral resolution, numerical stability, and computational tractability [50]. Additional details on the computational procedure are provided in [51].

### Appendix A.2. Rectified DMD Mode

For each 2 s segment, we run DMD on the stacked state in (A1) with stacking parameter *S* and truncation rank *R*, which yields the augmented mode matrix

$$\Phi_{\mathrm{aug}}=\left[\phi_{1}^{\mathrm{aug}},\ldots ,\phi_{R}^{\mathrm{aug}}\right]\in C^{(MS)\times R},\;M=19.$$

Since the augmented state stacks *S* consecutive *M*-channel snapshots, each $\phi_{j}^{\mathrm{aug}}$ consists of *S* channel blocks of length *M*. To report channel-level (physical) modes, we extract the first *M* entries of each augmented mode by the selection operator

$$\Pi =\left[\;I_{M}\;\;0\;\;\cdots \;\;0\;\right]\in R^{M\times (MS)},\;\Phi :=\Pi \;\Phi_{\mathrm{aug}}=\left[\phi_{1},\ldots ,\phi_{R}\right]\in C^{M\times R}.$$

The corresponding eigenfrequencies ${\left\{\omega_{j}\right\}}_{j=1}^{R}$ are defined as in (A2) and we sort the modes in nondecreasing order of $\omega_{j}$.

Let $A=|\Phi^{\mathrm{raw}}|\in R_{+}^{M\times R}$ and $\Theta =∠\Phi^{\mathrm{raw}}$ be the elementwise magnitude and phase. Define the columnwise (per–mode) means

$$\mu =\frac{1}{M}\;A^{⊤}1\in R^{R},$$

and apply the phase–preserving rectification operator R(·) columnwise:

(A3) $$\tilde{A}=\max\;\left(A-1\mu^{⊤},\;0\right),\;\Phi :=R\;\left(\Phi^{\mathrm{raw}}\right)\;=\;\tilde{A}⊙e^{i\Theta },$$

where the maximum is taken elementwise and ⊙ denotes the Hadamard (elementwise) product. To prevent certain DMD modes from being dominated by globally uniform amplitude components, we subtract the sensor-uniform (rank-1) component within each mode. This step is not intended as a physiological artifact-removal procedure but as a feature-conditioning operation that suppresses non-specific global magnitude effects—such as those arising from mechanical harmonics, broadly shared field components, or other stimulus-locked contributions—so that relative inter-sensor structure can be more clearly expressed.
